# Social Media Tools for the Development of Pre-Service Health Sciences Researchers during COVID-19 in Pakistan

**DOI:** 10.3390/ijerph19010581

**Published:** 2022-01-05

**Authors:** Muhammad Zaheer Asghar, Seema Arif, Javed Iqbal, Pirita Seitamaa-Hakkarainen

**Affiliations:** 1 Department of Education, University of Helsinki, 00014 Helsinki, Finland; 2 School of Doctorate, Education & ICT (e-Learning), Universitat Oberta de Catalunya, 08018 Barcelona, Spain; 3 Department of Education, University of Management and Technology, Lahore 54700, Pakistan; Drseema.arif@gmail.com; 4 School of Education, Guangzhou University, Guangzhou 510006, China; javed@e.gzhu.edu.cn

**Keywords:** social media tools, research competencies, health sciences, COVID-19, research completion

## Abstract

The development of health sciences researchers has immense significance during a pandemic to control, manage, and prevent future outbreaks of the disease. This study focused on the use of social media tools (SMT) among pre-service health sciences researchers to complement their research competencies (RCT) and research completion levels (RC) during COVID-19. This study used the Vitae research development framework (RDF) to measure research competencies as a mediator between the use of social media tools and research completion levels among post-graduate health sciences students. A cross-section survey research approach was adopted to collect data from the post-graduate students (n = 410) enrolled in health sciences departments at universities in Pakistan. The SmartPLS 3.3.8 software was used to analyze data through Partial least square structural equation modeling (PLS-SEM). The results revealed that different social media tools such as communication, information management, and multimedia have a direct influence on the research competencies of the pre-service researchers and have an indirect effect on the research completion levels. Health sciences institutions may devise social-media-based instructional strategies to develop post-graduate students’ research competencies, such as personal effectiveness, research governance, and research engagement, to help them compile their research and complete their degree program in time during an emergency.

## 1. Introduction

The social chaos of COVID-19 affected all segments of society, especially the education sector [1]. It had a drastic effect on the world economy. Higher education across the world has also turned topsy-turvy. Higher education is the source of the knowledge economy; therefore, its continuation was an enormous challenge. Hence, medical and health workers as well as students were at the forefront in the fight against COVID-19; their education, particularly their timely completion of research, has been drastically affected [2]. The education and research in health sciences during pandemics are of utmost importance [3] for two reasons: first, to prepare health researchers against the pandemic; and, second, for research that contributes to the prevention and cure of a pandemic. In Pakistan, the Higher Education Commission (HEC) advised all universities and higher education institutions to switch to an online format so that the continuation of education was not compromised. Online healthcare services through online- and social-media-based resources were already being practiced by health care professionals, and were further catalyzed due to COVID-19 [4]. This was the reason that health sciences institutions, students, teachers, and researchers intensively used all available information, communication, and technology (ICT) resources ranging from the World Wide Web to social networking sites for the continuation of their education and research during the pandemic crisis [5,6].

The term “Social Media” [7] refers to “the online technologies and practices that people use to share opinions, insights, experiences, and perspectives” [8]. ResearchGate, Mendeley, Google Scholar, LinkedIn, Academia, Facebook, Twitter, and Google+ were the SM sites most frequently used by graduate students for academic purposes [9]. According to a recent estimate, social media is becoming increasingly popular globally; more than 2.65 billion people were SM users, whereas this figure is expected to reach 3 billion by 2021 [10]. Such rapid growth in SM users indicates that SM has vast potential for knowledge sharing and networking [11]. SM use in health sciences is also extensive; customers of the health sector may use it to get better information about health services, get reviews on some therapeutic methods, or share their personal experiences about a health service [12]. Social media is connecting health sciences research with practice [13]. It is also helping the researchers connect for the creation and dissemination of knowledge. Though health sciences post-graduate students and faculty have been social media users [7], their use of SM increased and was used for communication with their supervisors, as well as locating their target samples for data collection [14]. Social media in health sciences for research and practice has become a trending research area during the COVID-19 pandemic [15].

There are few studies available that have focused on the usage of social media tools for research purposes, despite the increased significance of social media to academics [16]. Unlike previous research, this study has focused on the effect of the use of social media tools on the development of research competencies and research completion for health sciences students during COVID-19. Previous studies have mainly focused on the general public usage of social media [17,18] or general university students during the COVID-19 pandemic [19].

This study has added knowledge to the previous literature in many ways. First, it has categorized and measured the use of different social media tools such as communication, collaboration, information management, multimedia services, and general use of social media among health sciences students during the pandemic. Secondly, when face-to-face traditional classes were ceased due to the COVID-19 lockdown, this study has viewed the effect of different social media tools on the development of research competencies for health sciences pre-service researchers. Finally, this study is novel in its focus on the research completion process of post-graduate health sciences students during the pandemic. The education and training of the health sciences researchers became important during the pandemic to produce health professionals for the effective control and management of the pandemic crisis. This study would benefit health sciences education policymakers to devise social-media-based instructional strategies to continue education and research training of the post-graduate health sciences students during the crisis.

This study has posed the following research questions based on the above discussion:

RQ1. What is the effect of different social media tools on research completion among health sciences post-graduate students during the COVID-19 pandemic?

RQ2. Is there a connection between the use of social media tools and research competencies for health sciences post-graduate students in the completion of their research during the COVID-19 pandemic? 

After the introduction, Section 2 of this paper reviews the literature. Next, the study has proceeded with the development of a conceptual framework and hypotheses. Section 3 presents the research methodology, research approach, population and sample, questionnaire development, and pilot testing. Section 4 consists of the data analysis and findings of the study. Section 5 elaborates the findings with reference to previous studies. Finally, Section 6 contains the conclusions of the study, with implications and future research suggestions.

## 2. Literature Review

It is evident from the studies in low- and middle-income countries such as Latin America that the use of digital and e-resources have improved the quality of health sciences research, health delivery, and health systems [20]. Informatics health alliances of the researchers from Latin American countries with American researchers and other international stakeholders exist to enhance research and training among their networks [21]. These networks have objectives to promote short- and long-term education and training opportunities in health sciences informatics for international health, to introduce regional health sciences researchers with biomedical informatics, and to expand the networks in universities and research centers from north to south, south to north, and south to south. These centers were required to introduce programs ranging from short training to diploma and degree programs in health and informatics [22,23,24]. According to Blas and colleagues [25], health education should be complemented with the training courses in health informatics such as “Introduction to Biomedical Informatics”, “Data Representation and Databases”, “Mobile Health”, and “Security, Confidentiality, and Privacy.” It was also suggested that research topics such as the “evaluation of health information systems”, “policy in health informatics, interoperability, and standards”, “evidence-based decision making in informatics”, “rural telemedicine”, “mobile health”, “electronic health records”, “sequence analysis and gene finding”, “tele-education”, and “the analysis of cost-effectiveness in biomedical informatics” be prioritized.

Health sciences professionals from developing countries including Pakistan lack the opportunities to participate in proper training programs such as the Informatics Training for Global Health (ITGH) for capacity building in health informatics. Proper departments of medical education and information technology are also not available at the university level to develop the health-sciences-informatics-related workforce in Pakistan. Health sciences faculties in Pakistan were not ready to adopt dedicated online resources for e-learning and research during the COVID-19 pandemic. However, health sciences post-graduate students have access to the internet, and they felt comfortable utilizing web 2.0 and social media tools for their research work due to its use of ease, performance expectancy, and the availability of colleagues and mentors in their social networks [26].

Social media tools are important for the dissemination of knowledge and engagement of researchers in scientific communication [27]. It is now easier to create online information and approach researchers worldwide. Digitally accessible research in the form of books, articles, videos, and images has become a topic of discussion in health sciences professional circles on social network sites. The international market of medical technologies, drugs, and health sciences has rapidly embraced the virtual spaces of social media networks to promote their research, product, and services during the COVID-19 pandemic [4,27]. Web 2.0 technologies have more significant potential to connect health sciences students and pre-service researchers with their supervisors [28]; SM creates virtual spaces where they can have one-to-one meetings and direct feedback of their work instead of a passive review of the content, thus promoting trust and collaboration between the students and their supervisors. This combination of technology and pedagogy provides innumerable ways to innovate and create virtual spaces where adequate research supervision can occur [29]. Health sciences researchers, faculty, students, and journal editors are now connected through versatile open and closed social networking platforms for raising their concerns, learning, and conducting research.

### 2.1. Conceptual Framework

It is important to clearly define the scope of the study before presenting the research methodology. The study has focused on different types of social media tools, competencies, and research completion.

#### 2.1.1. Defining Social Media

The presence of users’ developed content and web 2.0 technologies are referred to as social media tools [16]. The term web 2.0 was defined in the early 2000s as a trending way of using the internet in a collaborative and participatory way, as well as the development and modification of the internet content by the internet users [16]. Blogs and wikis are some examples of the early usage of social media [30]; social media networking sites such as Myspace and Facebook were introduced later. These days, social media networking sites have shown more advancements by becoming more user-friendly for the creation and modification of user-generated content [31]. For example, traditional writing, reading, and reference publishing activities are becoming more common through social media [32], such as Google Docs (a web 2.0 service that provides features to collaborate, share writing with peers, colleagues, and friends, as well as to publish work online) and Mendeley (a reference management, open-source software with a versatile reference sharing option in groups). In this broader context, this study defines social media tools as a website, application, or web-based service that is comprised of user-generated content and web 2.0 characteristics of material sharing within a group of people or wide audiences [33]. It may include a variety of social media tools such as Zoom, Skype, and Google Meet video conferencing tools, social networking or microblogging sites such as Twitter and Facebook, or online sharing repositories such as Flicker [16]. Keeping in view a wide variety of social media tools, the current study has adopted a Duman [34] classification of social media tools: social media usage in general [7], such as Facebook, Tweeter, and WhatsApp; communication tools such as Zoom, Google Hangout, Webinar, and Skype; collaborative tools such as Wikipedia and Statpedia; multimedia tools such as image services, video, and audio services; and information management tools such as Monkey survey and Google Docs.

#### 2.1.2. Defining Research Competencies

The literature has presented a debate on the term ‘competency’ [35,36]. There are two identified practical meanings of the word ‘competency’ [37]. The first meaning is about the output of a person, which means, competent performance. The second meaning is related to input, being the ‘underlying characteristics of a person to obtain competent performance’. According to researchers [38], a professional may have a wide range of ‘competencies’, but may not be able to perform something with complete competence. Further, competencies are comprised of the available resources that an expert should be able to utilize appropriately to mobilize the resources to appropriately address the professional needs, which has been considered as the real meaning of being competent [39]. Therefore, the scope of this study defines competencies as the resources available for health sciences post-graduate students to complete their research during the COVID-19 pandemic.

There are different models available to assess the development stages of the researcher’s competencies [40]. Bent and colleagues [41] proposed a model which has classified a researcher’s development into seven stages. The model was named the “Seven Ages of Research” that was comprised of “postgraduate students at master’s level, postgraduate students at the Ph.D. level, contractual research staff, early budding researchers, established researchers, senior researcher, and expert level.” The focus of a pan European research network [42] EURAXESS classified the research career into three stages: the first stage includes education up to the Ph.D. level (R1); the second stage includes Ph.D. degree holders, but not independent researchers (R2); and the third stage is comprised of established researchers that carry out research independently. Rowley and McCulloch [43] gave a four-stage model of a researcher’s development, which included “Apprentice, Member, Expert and Leader” in a hierarchal progression. This study has utilized the term pre-service researchers for health sciences post-graduate students, which are those who were enrolled in masters/M.Phil and Ph.D. programs. Pre-service researchers are at the R1 level of EURAXESS classification and the member level of the classification by Rowley and McCulloch [43].

The Vitae researchers development framework (RDF) was aimed to develop professional researchers in the UK [40]. It was developed by the UK Research Innovation (UKRI) to improve the employability of researchers in academic and non-academic sectors [40]. The Vitae RDF’s development work was started in 2011 [44]. It acts as a reference document to prepare researchers and research policies. It is a circular model. The Vitae (2011) research framework was considered to measure research competencies of the graduate students with four major domains: personal effectiveness (PE), research knowledge and intellectual abilities (KI), research and governance (RG), and researcher’s engagement and influence (EI) to disseminate research [44]. The RDF’s four major domains have 12 subdomains that are further divided into 63 sub-categories. Vitae describes that the development of a researcher does not occur linearly. This framework also uses different lenses to meet the needs of different sectors such as information literacy, early research, teaching, well-being, intellectual ability, mental health, leadership, and so forth. The Vitae RDF is not only utilized by universities in the UK, but by many universities and industries in Europe, South Africa, Australia, and Japan [45]. This study has utilized the Vitae RDF competencies model with its four domains: personal effectiveness (PE), research knowledge and intellectual abilities (KI), research and governance (RG), and researcher’s engagement and influence (EI) to measure the research competencies of the health sciences post-graduate students.

### 2.2. Hypothesis Development

Health sciences researchers [46,47] acknowledge that medical education and instructional technologies programs can help in knowledge dissemination to the mass number of trainees to update their knowledge, skills, and abilities through the use of e-resources, digital technologies, web 2.0, and social media. It would also enhance the capabilities of health policymakers as well as strategic planners at different levels by inculcating leadership and management skills in research, evaluation studies, and publications [48]. Social media was taken as an exogenous construct in this study that is hypothesized to influence research competencies, which is an endogenous construct of the study. According to research findings [49], the information and communication technologies, especially new media, play an important role in the development of research competencies among doctoral students., as evidenced by the increased utilization of social media tools among researchers during the COVID-19 pandemic [50]. Therefore, hypotheses were developed as follows:

**Hypothesis** **1.** *The use of social media tools significantly and positively influenced the research competencies of the post-graduate health science students during the COVID-19 pandemic.*

**Hypothesis** **1.1.** *The use of social media, in general, significantly and positively influences research competencies.*

**Hypothesis** **1.2.** *Communication tools significantly and positively influence research competencies.*

**Hypothesis** **1.3.** *Collaborative tools significantly and positively influence research competencies.*

**Hypothesis** **1.4.** *Information management tools significantly and positively influence research competencies.*

**Hypothesis** **1.5.** *Multimedia tools significantly and positively influence research competencies.*

Training programs related to medical informatics may improve international linkages of e-health centers around the globe through the use of web 2.0 and social media. It would result in effective research outcomes on e-health-related topics such as mobile health, the internet of things, artificial intelligence, digital repositories, big data, and social and cross-cultural health issues [51]. It is thought that social media helps scholars and faculty members to develop a connection with other researchers [16], which results in the timely completion of their research during the crisis [52], as social media played a role in connecting post-graduate students with colleagues, teachers, and institutions to compile their research tasks [53]. This study has also considered social media as an exogenous construct that influences research completion, which is assumed to be an endogenous construct. The hypothesis was developed as follows:

**Hypothesis** **2.** *The use of social media tools significantly and positively influenced the research completion levels of health sciences post-graduate students during the COVID-19 pandemic.*

**Hypothesis** **2.1.** *Social media use, in general, significantly and positively influences research completion.*

**Hypothesis** **2.2.** *The use of communication tools significantly and positively influences research completion.*

**Hypothesis** **2.3.** *The use of collaborative tools significantly and positively influences research completion.*

**Hypothesis** **2.4.** *The use of information management tools significantly and positively influences research completion.*

**Hypothesis** **2.5.** *The use of multimedia tools significantly and positively influences research completion.*

Research competencies for researcher’s development are defined as knowledge, engagement, management, and effectiveness [44]. Moreover, the essential factors for the completion of research tasks have been defined as a set of research skills, research management skills, communication skills, funding skills, and knowledge dissemination skills [44,54]. The research competencies that are developed during the students’ post-graduate research help them to become future independent researchers [41]. The studies have found a positive relationship between research competencies and research productivity [55]. Studies have reported that a researcher’s low level of research competencies results in fewer chances of research completion [56,57]. Based on this argument, the following hypothesis was developed to assess the relationship between research competencies and research completion levels:

**Hypothesis** **3.** *The research competencies of health sciences post-graduate students influence the completion of their research.*

The use of information and new technologies in the development of research competencies is a part of the pre-service researcher’s professional development. Empirical studies from middle-income countries have found that the use of digital resources has shown enormous benefits in improving the strategic planning process by providing access to e-health content, linking health professionals and researcher’s networks, and improving case detection processes [48,58]. Research competence enhances research productivity among students [59]. Social media tools provide help to develop research competencies among health education students, which ultimately enhances their research completion process [60]. According to researchers, the idea of research competencies is considered as the ability to gain knowledge, skills, and attitude to use ICT resources, such as the internet and social media, which ultimately help in developing, planning, organizing, conducting, and enhancing the research completion procedure [61]. Similarly, social media use by researchers during the COVID-19 pandemic has proven to be a catalyzing agent to improve research skills and enhance research output [59]. Based on this argument, a relationship between social media tools and the research process, mediated by research competencies, was assumed in the following hypotheses:

**Hypothesis** **4.** *There is a meditating role of research competencies between the use of social media tools and research completion level during the COVID-19 pandemic among post-graduate health sciences students.*

**Hypothesis** **4.1.** *Research competencies mediate the relationship between the general use of social media and research completion.*

**Hypothesis** **4.2.** *Research competencies mediate the relationship between the use of communication tools and research completion levels.*

**Hypothesis** **4.3.** *Research competencies mediate the relationship between collaborative tools and research completion levels.*

**Hypothesis** **4.4.** *Research competencies mediate the relationship between the use of information management tools and research completion levels.*

**hypothesis** **4.5.** *Research competencies mediate the relationship between the use of multimedia tools and research completion levels.*

**Figure 1:**
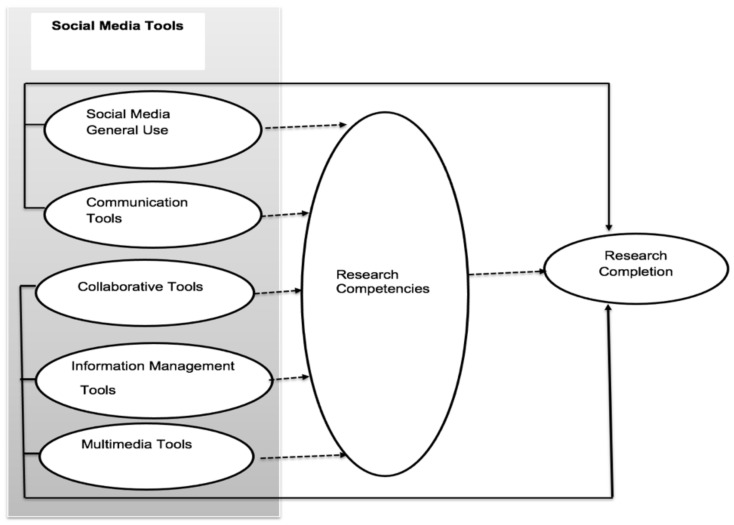
Conceptual framework.

A visual depiction of the hypothesized model is shown in Figure 1.

## 3. Research Method

### 3.1. Research Approach

This study used a cross-sectional survey research approach to collect the data on social media tools, research competencies, and the research completion process from the students enrolled in health science programs in the universities of Pakistan. The survey approach was considered helpful for three reasons. First, the influence of social media tools used on the development of research competencies and the research completion process among students is a phenomenon that required a self-reported survey to measure the respondents’ experience during COVID-19. Second, data collection from a large sample would be helpful to generalize the results to the population. Finally, the researcher would add a professional link to access respondents during the COVID-19 pandemic. A survey questionnaire (instrument) was developed to collect the data.

### 3.2. Instrumentation

The questionnaire was initially developed by Duman [34] and modified by the researchers to measure the usage of social media tools. The questionnaire was comprised of three sections. Section 1 introduced the purpose of the study. It sought the participants’ consent for their volunteer participation. Additionally, it presented the information related to the confidentiality, anonymity, data protection procedures, and demographic profile of participants (gender, age, university type). Section 2 contained survey items related to social media tools, research competencies, and the research completion process, measured on a 5-point Likert scale ranging from 1 = strongly agree to 5 = strongly disagree. A pilot test was conducted on ten graduate and twenty post-graduate scholars enrolled in health sciences programs with similar characteristics. Constructs, sub-constructs, and their relevant indicators are provided in Supplementary Material Table S1.

### 3.3. Constructs Measurements

#### 3.3.1. Social Media General Usage

Six items related to the general usage of social media were adapted from the work of Duman [34]. Students were asked about their social media usage in general [34]. The sample items are as follows: “social media is part of my everyday activity”, “I am proud to tell people that I use social media”, “social media has become a part of my daily routine”, and “I feel I am part of an online community”. The reliability of the social media’s general usage construct was found to be α = 0.90.

#### 3.3.2. Communication Tools

Eight items related to communication tools were adapted from the work of Duman [34]. The construct of social-media-based communication tools [34] was acquired through rating the use of following resources for research purposes: social network sites such as Facebook and Twitter, academic and social network sites such as Academia.edu and ReseachGate, WhatsApp/instant messaging, internet discussion forums, mailing lists, blogging, microblogging, and RSS feeds. The reliability of the communication tools was found to be α = 0.95. Therefore, it was concluded that the scale was suitable for final data collection.

#### 3.3.3. Collaboration Tools

Four items related to collaboration tools were adapted from the work of Duman [34]. The construct of social media tools for collaboration [34] was investigated through the determination of the items that students used most in each category, being collaborative writing resources, video conferencing, social bookmarking, and Wikis. The reliability of the collaboration tools was found to be α = 0.93.

#### 3.3.4. Information Management Tools

Students were further asked about their social media usage for research management [34] under the following categories: citation or reference management resources, e-information or academic database, online library catalog, survey resources, learning management systems, and project management. The reliability of the collaboration tools was found to be α = 0.96.

#### 3.3.5. Multimedia Tools

Students were asked about the variety of multimedia tools of social media for academic purposes [34] under the following categories: presentation services, video services, photo services, file services, and audio podcasting. The reliability of the multimedia tools was found to be α = 0.96.

#### 3.3.6. Research Competencies

The construct of research competencies was divided into sub-factors as follows:

##### Information Management Tools

Students were further asked about their social media usage for research management [34] under the following categories: citation or reference management resources, e-information or academic database, online library catalog, survey resources, learning management systems, and project management. The construct’s reliability was found to be satisfactory at α = 0.96.

##### Personal Effectiveness

Students were asked to rate the development of their personal research skills [34,44] for the following categories: career management, continuing professional development, academic networking, academic reputation and esteem, work-life balance, time management, and preparation and prioritization. The construct’s reliability was found to be satisfactory at α = 0.88.

##### Research Knowledge and Intellectual Abilities

Students were asked to rate their enhancement in their research knowledge and intellectual abilities [34,44] for the following categories: subject knowledge, theoretical knowledge on research methods, practical application on research methods, information seeking, information literacy and management, academic reading, critical thinking, and problem solving. The construct’s reliability was found to be satisfactory at α = 0.93.

##### Research Governance and Management

Students were asked to rate their improvement in their research management skills [34,44] for each category: research management, multimedia management, reference management, financial management, seeking funding, and seeking a scholarship. This construct’s reliability was found to be satisfactory at α = 0.89.

##### Engagement, Influence, and Impact

Students were asked to rate their engagement in research activities and their relative influence and impact on research productivity [34,44] under the following categories: publication, presentations at conferences, communication, collaboration, team working, people management, supervision, and teaching. The construct’s reliability was found to be satisfactory at α = 0.92.

#### 3.3.7. Research Completion Level

The construct of research completion [34] asked students about how many research tasks have they compiled, including generating ideas, background work, preparing and organizing, collecting data, analyzing, writing, creating, revisiting primary research output, and defending their research project or thesis. The reliability of the research completion levels was found to be α = 0.97, which showed that the scale was suitable for final data collection.

### 3.4. Data Collection

Pre-service health sciences students enrolled in graduate and post-graduate programs in the universities located in Pakistan were the target population of this study. An online calculator [62] helped us to find the required sample size for research that utilizes partial least square structural equation modeling. This study used 37 latent variables and 7 observed variables in the model, the anticipated effect size was 0.25, and the desired statical power was 0.8 at a probability level of 95%. The calculator gave suggestions based on statistical formulae [63,64] that the minimum sample size should be 397 to generalize the results on population.

A stratified sampling technique was used to select data from 15 public universities and 15 private universities that were randomly selected from all universities located in Pakistan. The online survey questionnaire was prepared and sent to the randomly selected (Masters, n = 400; Ph.D., n = 200) email addresses of the students. A total number of 410 health sciences post-graduate students responded to the survey. The respondents were comprised of: 207 (50.5%) male and 203 (49.5) females; 186 (45.4%) respondents were from private universities and 224 (54.7%) were from public sector universities; 276 (67.4%) respondents were MPhil students while 134 (32.8%) were Ph.D. students; 184 (44.8%) respondents were less than 29 years of age, 159 (38.7%) were between 25–30 years old, 42 (10%) were between 30–35 years old, 9 (2.2%) were between 35–40 years old, 11 (2.8%) were between 40–45 years old, and 6 (1.4%) respondents were above 45 years.

## 4. Data Analysis

The data were entered into SPSS (IBM, Armonk, NY, USA) software. Data screening was performed for outliers and missing values. The robustness of the data was checked for heterogeneity, endogeneity, and non-linearity. We applied partial least square structural equation modeling (PLS-SEM) for data analysis for three reasons: first, [65] PLS-SEM is helpful for multivariate analysis to measure the cause and effect between the exogenous and endogenous constructs; second, PLS-SEM aided in testing complex multivariate models for the exploration and development of new theoretical aspects [66,67]; and third, PLS-SEM can be applied for formative constructs. Due to the complexity of the conceptual model, the SmartPLS 3.2.8 (SmartPLS GmbH, Bönningstedt, Germany) software was found to be the most appropriate to check the cause–effect relationships among the constructs [68]. PLS-SEM was employed to assess the effect of the usage of social media tools on the development of research competencies and the timely research completion process. Most importantly, the construct of research competencies has four dimensions. A second-order factor analysis was performed to combine four formative indicators, such as research engagement and influence (EI), knowledge and intellectual abilities (KI), personal effectiveness (PE), and research governance (RG), to develop a formative construct of research competencies (RC). Therefore, since it was a theory exploration process, PLS-SEM was found to be most suitable for this study. Following the guidelines of previous research, a two-step analysis of the data was conducted [69]. First, a measurement model was evaluated to measure the reliability and validity of the constructs [70]. Next, the structural model was evaluated for the cause-effect relationships, specific indirect paths, effect size, and coefficient of determination [71].

### 4.1. Measurement Model Evaluation

The guidelines set forth by Ho [72] were followed for assessing the outer model for construct reliability. First, single item consistency was observed, and then the relevance of the unobserved construct was checked in contrast to the observed items. The items with an alpha score above the threshold of 0.6 were accepted to make up a relevant construct [73,74]. All items included had factor loadings above 0.7. Convergent validity was measured through average variance extracted (AVE) values. The set standard is to meet the threshold of 0.5, which was met adequately. The composite reliability value (CR) also met the criterion threshold of 0.7 [75]. The instrument was evaluated at two levels. The first-order analysis was performed for the confirmatory factor analysis (CFA) of the primary factors. The reliability α > 0.7, rho > 0.7, AVE > 0.5, and CR > 0.7 were found satisfactory for all factors. See Table 1 for further details.

This study has used the latest approach for measuring discriminant validity through HTMT (heterotrait–monotrait correlation ratios). Hansler and colleagues [76] proposed that the HTMT value below index 1 is valid. We observed the HTMT values for all constructs below 0.9 and satisfactory [77], as given in Table 2.

#### Second-Order Factor Analysis

The second-order factor analysis was performed to combine the sub-factors of research competencies as formative indicators. Hair and colleagues [78] suggested that a formative indicator should have an outer weight level minimum of 0.5, or it must have significant loading to become part of the main construct in higher-order factor analysis. The outer weights were significant for all sub-factors of research competencies except knowledge and intellectual abilities (*p* > 0.05), while all formative indicators have shown significant outer loading for the formative construct of research competencies. Previous research [79] suggested that the variance inflation factor (VIF) must be less than the threshold of 3.3 for a satisfactory level of multicollinearity. We found all VIF values to be less than 3.3, which negates collinearity issues for formative indicators combined to form a formative construct, as shown in Table 3.

### 4.2. Structural Model Evaluation

The structural equation model was assessed for the exogenous construct’s effect size on endogenous variables, adjusted R^2^, Stone–Geiser’s predictive relevance (Q-square) of the endogenous variables, and direct and specific indirect paths.

#### 4.2.1. Goodness of Fit Model

The standardized root mean residual (SRMR), which is an index between hypothesized covariance and observed matrices [80], was used to measure the model fitness. SRMR values less than the threshold of 0.8 are satisfactory for model fitness, while NFI values should be above the threshold of 0.8. Another set of criteria to measure the model fitness, rms_theta, calls for a threshold below 0.12. The model was a good fit with SRMR = 0.039, NFI = 0.953, and rms_theta = 0.11; see Table 4 below.

All constructs have shown an inner VIF value below 0.5, showing no multicollinearity issues between the constructs [81]. See Table 5 below.

#### 4.2.2. Coefficient of Determination

The coefficient of determination, R^2^_,_ indicates a change in the dependent variables for the per unit change in the independent variable. The values of R^2^ greater than 0.01 were accepted. See Table 6.

#### 4.2.3. Effect Size

The degree of the effect of the exogenous construct on the endogenous construct is measured with f^2^. The effect size is considered weak if the value is below 0.02, is considered moderate with a value of 0.15, and is considered substantial with a value above 0.35 [82]. The summary of the endogenous constructs with their effect size is given in Table 7.

#### 4.2.4. Redundancy Analysis

Besides the R^2^ values, we measured the predictive criterion accuracy with Stone–Geisser’s Q^2^ value [83], which assesses the quality of the model. Q-square is measured with blindfolding in PLS-SEM, reflecting the predictability of the endogenous constructs. A cross validity redundancy analysis yielded the value of Q^2^ (=1–SSE/SSO), greater than zero, which is acceptable for endogenous constructs in SEM; See Table 8.

#### 4.2.5. Direct Path

**Figure 2:**
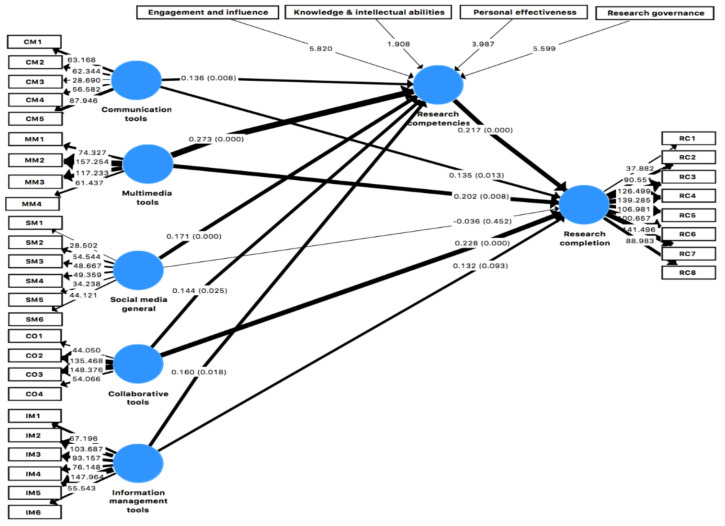
Path analysis.

The mean path *β* coefficient for regression values was used in PLS-SEM to test the hypotheses [84]. The *β* indicated the per unit change effect of the independent construct on the dependent construct, whereas the significance values and t-statistics verified the *β* using bootstrapping [85]. The path coefficient values, significance level, and t-statistics with a bootstrapping of 5000 sub-samples are shown in Table 9. The relationships extracted through the path coefficient and specific indirect effects are illustrated in Figure 2.

First, we analyzed the results for hypothesis 1, “Social media tools positively influence research completion levels”, which was divided into sub hypotheses to measure the effect of different social media on research completion levels. Social media, in general, did not influence research completion levels (*p* > 0.05). Hence, hypothesis 1.1 was rejected. Communication tools positively influence research completion (*β* = 0.136, t = 2.475, *p* = 0.013). Hence, hypothesis 1.2 was accepted. Collaborative tools influence the research completion levels (*β* = 0.228, t = 3.569, *p* = 0.000). Hence, hypothesis 1.3 was accepted. Information management tools did not influence research completion levels (*p* > 0.05). Hence, hypothesis 1.4 was rejected. Multimedia tools significantly influence research completion levels (*β* = 0.202, t = 2.649, *p* < 0.000). Hence, hypothesis 1.5 was accepted, as given in Table 9.

Second, we analyzed the results for hypothesis 2 “Social media tools positively influence research competencies”. We divided this hypothesis into sub hypotheses as we classified social media into different categories such as collaborative tools, communication tools, information management tools, multimedia tools, and social media usage in general. Social media usage in general influenced research competencies of the pre-service health sciences students (*β* = 0.171, t = 3.486, *p* = 0.000). Hence, hypothesis 2.1 was accepted. Communication tools influenced research competencies significantly (*β* = 0.139, t = 2.658, *p* = 0.008). Hence, hypothesis 2.2 was accepted. Social media collaborative tools significantly influenced the research competencies of the pre-service health sciences students (*β* = 0.144, t = 2.234, *p* = 0.025). Hence, hypothesis 2.3 was accepted. Information management tools influenced research competencies significantly (*β* = 0.160, t = 2.371, *p* = 0.018). Hence, hypothesis 2.4 was accepted. Multimedia tools significantly influenced research competencies (*β* = 0.273, t = 4.712, *p* = 0.000). Hence, hypothesis 2.5 was accepted, as given in Table 9.

Research competencies have shown a positive and significant influence on research completion (*β* = 0.222, t = 4.208, *p* = 0.000). Hence hypothesis 3 was accepted as given in Table 9.

#### 4.2.6. Specific Indirect Paths

Hypothesis 4 was tested for the mediation of research competencies between the use of social media tools and research completion. The main hypothesis was divided into sub hypotheses based on different forms of social media tools. Research competencies showed a positive mediation between social media use in general and research completion levels (*β* = 0.037, t = 2.359, *p* = 0.018). Hence, hypothesis 4.1 was accepted. Research competencies showed a positive mediation between communication tools and research completion levels (*β* = 0.029, t = 2.293, *p* = 0.022). Hence, hypothesis 4.2 was accepted. Research competencies did not show mediation between collaborative social media tools and research completion levels (*p* > 0.05). Hence, hypothesis 4.3 was rejected. Research competencies showed a positive mediation between information management tools and research completion levels (*β* = 0.035, t = 2.08, *p* = 0.038). Hence, hypothesis 4.3 was accepted. Research competencies showed a positive mediation between multimedia tools and research completion levels (*β* = 0.059, t = 3.112, *p* = 0.002). Hence, hypothesis 4.4 was accepted as given in Table 10.

## 5. Discussion

This research endeavored to explore the relationship between different social media tools used on research competencies and research completion of the health sciences students at the post-graduate level during the COVID-19 pandemic. Previous research has mostly explored the effect of e-resources on research competencies. To the researcher’s best knowledge, this is the first study that measured the mediation of research competencies between different social media tools and research completion levels during the COVID-19 crisis.

First, this study explored the direct association between social media tools and the research completion levels. The results showed three out of the five dimensions of social media tools, including communication tools, collaborative tools, and multimedia tools, have a positive and significant relationship with the research completion levels. In contrast, two out of five dimensions of social media tools, including general social media use and information management tools, have an insignificant relationship with the research completion process. As such, the data supports hypotheses H1.2, H1.3, and H1.5 while H1.1 and H1.4 are rejected. The results of the present study are consistent with previous studies [27]. According to previous research [86], the use of Wikis, blogs, and regular engagement on social media for research helps health sciences students to compile their research tasks. Zoom and other video conferencing tools such as Google Meet and Skype helped health sciences post-graduate students to share information and knowledge during the COVID-19 pandemic [87]. However, some scholars do not positively view SM usage; they claim students get distracted and lose focus, negatively affecting their productivity [88]. Others argue that too much SM usage causes an addiction, deteriorating students’ mental well-being [8]. Social media was an essential source of communication during COVID-19 and, therefore, research students get the benefit of it for communication, data collection, and collaboration to complete their research.

Second, the current research found a positive and significant influence of different social media tools such as communication, collaboration, information management, multimedia, and general social media usage on the development of research competencies for pre-service health sciences researchers. The study findings have elaborated that SM usage positively influenced research competencies, such as enhancing research engagement, research governance, and personal influence. Hence, hypotheses H2.1, H2.2, H2.3, H2.4, and H2.5 were approved. These findings are congruent with previous research [89,90]. The research engagement of the students was increased because of SM usage for networking and disseminating information, as well as developing public awareness about recent research developments [91]. SM usage was also beneficial for the improvement of decision-making through bibliometrics measurement, an essential aid for research governance [92]. Researchers can use social media such as blogs and ResearchGate to circulate their research results to enhance their influence on society [93]. During the COVID-19 pandemic, social media tools were widely available sources for research that helped health sciences post-graduate students to improve their research competencies.

Third, this study found a positive and significant effect of research competencies on the research completion process of the pre-service health sciences researchers. Hence hypothesis 3 was approved. The finding of this research is aligned with previous studies [59,94]. According to previous research [95], higher education institutions need to impart high-level research knowledge and skills in post-graduate students to produce better research outcomes. Research competencies are required under normal circumstances, as well as during the pandemic crisis, in order to complete the research process.

Fourth, our study noticed that the research competencies of the pre-service health sciences researchers mediated the effect between the usage of different social media tools, except collaborative tools, on the research completion process during the COVID-19 pandemic. Hence, hypotheses H4.1, H4.2, H4.4, and H4.5 were approved and hypothesis H4.3 was rejected. According to the findings of the present study, collaborative social media tools like Wikipedia were not helpful for the development of research competencies and the research completion process. These findings align with previous research; it is held that, although collaborative social media sites like Wikipedia are unreliable, they can serve as a source of initial information, but do not play any significant role in content development or research completion [96]. Pre-service health sciences researchers must be mindful of this reality while using collaborative SM tools. The other results also align with previous research that information sharing and knowledge sharing increased during COVID-19 [97]; graduate students used the SM sites worldwide to improve their research competencies [59]. Previous research [98] authenticated that information management tools, such as Google Docs, helped gather and manage telework during COVID-19. Pre-service researchers used information management tools, such as Monkey surveys and Google Docs, for data collection during the pandemic, confirming the findings of previous studies [99], whereas information management tools such as Google Docs and Monkey surveys, proved valuable for data collection during COVID-19. Students have taken advantage of SM tools liberally in their dissertation writing process; they have been using various tools including reference management, academic databases, online library catalogs, and file services. The findings related that social media communication tools such as Zoom, Google Hangout, and webinars were beneficial in developing research competencies and completing research tasks rallied with other research. SM usage has impacted the development of research competencies and scholarly communication during COVID-19 [100], which resulted from their research completion. Pre-service researchers used SM tools, Skype, and Zoom extensively for communicating with their supervisor, for data collection, and especially for interviews [101]. Moreover, they were enthusiastic about discovering new techniques and methods to develop their competencies and enhance their research quality. The study findings established that multimedia tools such as video, audio, and image transfer services have extensively influenced the development of research competencies, leading to the research completion of pre-service health sciences researchers. Mastery in multimedia usage is an essential part of the health sciences curriculum, and multimedia tools have a wide presence at social media sites. These findings are in sync with other research results, revealing that audio, video, digital, and social media tools affect the language competencies of student researchers, familiarizing them with the research diction [102]. Networking with other researchers on SM sites, such as Facebook and Twitter, leaves a more substantial effect in this respect than general SM usage. A Malaysian study concluded that SM usage enhances pre-service researchers’ research skills by research engagement and collaboration with faculty and peers [90]. General usage of SM and information management tools were not found to affect timely research completion in the primary evaluation of the model; however, their influence in the timely completion of research was confirmed by the presence of research competencies.

## 6. Conclusions

SM usage was found to be beneficial for the development of research competency and for the completion of mandatory research tasks for Pakistani pre-service health sciences researchers during COVID-19. The student researchers had indulged in the general and research-specific use of SM tools during the pandemic. Social media sites such as Facebook, WhatsApp, and Instagram, were advantageous for networking, and communication tools, such as Skype and Zoom, were used for information gathering. Collaborative tools such as Wikipedia and Statpedia were also helpful for them. Information management tools such as Google Docs and Survey Monkey were valuable sources for data collection during COVID-19. It is safely concluded that smart usage of SM tools can build research competencies among pre-service health sciences researchers, transforming them into knowledge producers. This transformation would help to build the knowledge economy, especially, the needs of health sector development in Pakistan. Therefore, ICTs integration can be safely ensured through the usage of SM tools among pre-service health sciences researchers to develop sustainable health delivery services and systems of the country. The results would draw the attention of health education faculty, managers, and policymakers towards the implication of the issue of social media integration for researchers’ development.

### Implications

The pre-service health sciences researchers used social media such as Facebook, WhatsApp, and Instagram to link with their class fellows, teachers, and other researchers. Worldwide, governments have partnered with WhatsApp to provide updated information about COVID-19 [103]. The Pakistan health ministry and many other health agencies also provided ‘real-time’ data about public health and COVID-19 spread on Facebook and Twitter, which was of great concern for health sciences researchers; explicated policies and implementation are needed in this respect.

Knowledge sharing is one of the effective strategies for graduate research. SM has infiltrated academic life profusely; therefore, it is time to harness this energy [104]. Faculty and students must exploit the SM resources to achieve excellence in health education systems by creating informal chat groups within intra-student, student-faculty, and interfaculty groups for meeting research and study goals.

Graduate medical education is not free and is costly to its customers; developing countries and universities have limited resources to expand their research resources. The research is a much-desired product for a university’s health sciences reputation and provides higher worldwide rankings [105]. Timely completion of the degree is obligatory in Pakistan; otherwise, its cost may rise inexplicably. Mainly, in the COVID-19 scenario, the pre-service researchers who could connect with their supervisors could continue their research. It was not a one-time situation; living in turbulent times, the universities must gear up their staff and students to meet such challenges in the future. Health sciences institutions themselves are slowly moving towards emergent technologies for online and blended learning through social media networks.

## Figures and Tables

**Table 1:** First-order constructs reliability and validity.

First-Order Reflective Constructs	Items	Loadings	α	Rho_A	CR	AVE
Research completion level (RC)	CL1	0.844	0.97	0.98	0.98	0.84
	CL2	0.912				
	CL3	0.93				
	CL4	0.935				
	CL5	0.924				
	CL6	0.926				
	CL7	0.934				
	CL8	0.914				
Collaborative tool (CO)	CO1	0.854	0.93	0.93	0.95	0.82
	CO2	0.939				
	CO3	0.932				
	CO4	0.896				
Communication tools (CM)	CM1	0.776	0.94	0.95	0.95	0.72
	CM2	0.849				
	CM3	0.701				
	CM4	0.892				
	CM5	0.869				
	CM6	0.895				
	CM7	0.891				
	CM8	0.878				
Engagement and Influence (EI)	EI1	0.849	0.92	0.93	0.94	0.65
	EI2	0.785				
	EI3	0.787				
	EI4	0.791				
	EI5	0.812				
	EI6	0.751				
	EI7	0.85				
	EI8	0.81				
Information management tools (IM)	IM1	0.884	0.96	0.96	0.97	0.83
	IM2	0.923				
	IM3	0.907				
	IM4	0.922				
	IM6	0.938				
	IM6	0.897				
Knowledge and intellectual abilities (KI)	KI1	0.767	0.93	0.93	0.94	0.71
	KI2	0.868				
	KI3	0.803				
	KI4	0.867				
	KI5	0.834				
	KI6	0.907				
	KI7	0.828				
Multimedia tools (MM)	MM1	0.899	0.96	0.96	0.97	0.85
	MM2	0.929				
	MM3	0.937				
	MM4	0.932				
	MM5	0.911				
Personal effectiveness (PE)	PE1	0.778	0.88	0.88	0.91	0.59
	PE2	0.716				
	PE3	0.767				
	PE4	0.837				
	PE5	0.783				
	PE6	0.83				
	PE7	0.648				
Research governance (RG)	RG1	0.864	0.89	0.90	0.92	0.65
	RG2	0.732				
	RG3	0.847				
	RG4	0.858				
	RG5	0.837				
	RG6	0.676				
Social media general use (SM)	SMG1	0.771	0.90	0.91	0.93	0.67
	SMG2	0.845				
	SMG3	0.843				
	SMG4	0.843				
	SMG5	0.791				
	SMG6	0.823				

**Table 2:** Discriminant validity.

	CL	CM	CO	IE	IM	KI	MM	PE	RG
CL									
CM	0.703								
CO	0.718	0.757							
EI	0.577	0.545	0.608						
IM	0.674	0.702	0.755	0.63					
KI	0.559	0.512	0.513	0.644	0.534				
MM	0.721	0.78	0.811	0.63	0.792	0.544			
PE	0.574	0.649	0.588	0.579	0.609	0.73	0.672		
RG	0.627	0.629	0.607	0.672	0.6	0.706	0.662	0.757	
SM	0.496	0.563	0.529	0.547	0.682	0.512	0.629	0.546	0.517

Research completion level (RC); Collaborative tool (CO); Communication tools (CM); Engagement and Influence (EI); Information management tools (IM); Knowledge and intellectual abilities (KI); Multimedia tools (MM); Personal effectiveness (PE); Research governance (RG); Social media general use (SM).

**Table 3:** Formative measurement model.

Formative Construct	Indicators	Significance of Outer Loading			Significance of Outer Weight			VIF
		Loading	t-Stats	*p* Values	Weight	t-Stats	*p* Values	
RCT	EI	0.858	22.874	0.000	0.424	5.731	0.000	1.803
	KI	0.780	20.309	0.000	0.121	1.889	0.059	2.358
	PE	0.817	28.521	0.000	0.270	3.932	0.000	2.384
	RG	0.874	33.548	0.000	0.360	5.503	0.000	2.273

Research competencies (RCT); Engagement and Influence (EI); Knowledge and intellectual abilities (KI); Personal effectiveness (PE); Research governance (RG).

**Table 4:** Model fit criteria.

Fit Values	Saturated Model	Rms_Theta
SRMR	0.039	0.11
NFI	0.953	

**Table 5:** VIF values.

	Research Competencies	Research Completion
Social media-general	1.777	1.844
Collaborative tools	2.855	2.904
Communication tools	2.031	2.073
Information management tools	3.102	3.16
Multimedia tools	3.638	3.81
Research competencies		2.31

**Table 6:** Coefficient of determination.

Endogenous Constructs	R Square	R Square Adjusted
Research competencies	0.567	0.562
Research completion	0.593	0.587

**Table 7:** Effect Size.

Constructs	Research Competencies	Research Completion
Social media-general	0.038 (moderate)	0.002 (weak)
Collaborative tools	0.017 (weak)	0.044 (moderate)
Communication tools	0.021 (moderate)	0.022 (moderate)
Information management tools	0.019 (moderate)	0.013 (weak)
Multimedia tools	0.047 (moderate)	0.026 (moderate)
Research competencies		0.05 (moderate)

**Table 8:** Cross Validity Redundancy Analysis.

	SSO	SSE	Q^2^ (=1–SSE/SSO)
Research competencies	1760	1078.844	0.387
Research completion	3520	1788.27	0.492

**Table 9:** Direct paths.

Hypotheses	*β*	t Stats	*p*	Status
Social media General → Research completion	−0.036	0.753	0.452	rejected
Communication tools → Research completion	0.136	2.475	0.013	Accepted
Collaborative tools→ Research completion	0.228	3.569	0.000	Accepted
Information management tools → Research completion	0.132	1.679	0.093	rejected
Multimedia tools → Research completion	0.202	2.649	0.008	Accepted
Social media general →Research competencies	0.171	3.486	0.000	Accepted
Communication tools → Research competencies	0.139	2.658	0.008	Accepted
Collaborative tools→ Research competencies	0.144	2.234	0.025	Accepted
Information management tools → Research competencies	0.160	2.371	0.018	Accepted
Multimedia tools → Research competencies	0.273	4.712	0.000	Accepted
Research competencies → Research completion	0.222	4.208	0.000	Accepted

**Table 10:** Specific Indirect paths.

Hypotheses	*β*	t	*p*	Status
Social media general → Research competencies → Research completion	0.037	2.359	0.018	Accepted
Communication tools → Research competencies → Research completion	0.029	2.293	0.022	Accepted
Collaborative tools → Research competencies →Research completion	0.031	1.785	0.074	Rejected
Information management tools → research competencies → research completion	0.035	2.08	0.038	Accepted
Multimedia tools → research competencies → research completion	0.059	3.112	0.002	Accepted

## Data Availability

The datasets used and/or analyzed during the current study are available from the corresponding author upon reasonable request.

## Supplementary Materials

The following supporting information can be downloaded at: https://www.mdpi.com/article/10.3390/ijerph19010581/s1, Table S1: Main constructs, sub-constructs and indicators.
