# Supplementary material for: Social Media Tools for the Development of Pre-Service Health Sciences Researchers during COVID-19 in Pakistan
Source: Int J Environ Res Public Health. 2022 Jan 5;19(1):581. doi: 10.3390/ijerph19010581 (PMC8744709; doi:10.3390/ijerph19010581)
Supplement: Supplementary file 1 [file ijerph-19-00581-s001.zip › ijerph-1494583-supplementary.pdf]

Supplementary material

Table S1. Main constructs, sub-constructs and indicators

Main Constructs	Sub Constructs	Indicators
Social Media Tools	Social media use in general	Social media is part of my everyday activity<br>I am proud to tell people that I use social media<br>Social media has become a part of my daily routine<br>I feel proud that I am part of an online community<br>I visit my social media accounts<br>I keep updating my social media profiles
	Communication Tools	Resources for research purposes social network sites such as Facebook, Twitter.<br>Academic and social network sites like Academia.edu and ReseachGate,<br>WhatsApp/ instant messaging,<br>internet discussion forums,<br>Mailing list<br>Blogging<br>Microblogging<br>RSS feeds
	Collaborative Tools	Collaborative writing resources<br>Video conferencing<br>Social bookmarking<br>Wikis
	Multimedia tools	Presentation services<br>Video services<br>Photo services<br>File services<br>Audio podcasting
	Information management tools.	Citation or reference management resources<br>E-information or academic database<br>Online library catalog<br>Survey resources<br>Learning management systems<br>Project management.
Research Competencies	Personal effectiveness.	Career management,<br>Continuing professional development<br>Academic networking,<br>Academic reputation and esteem,<br>Work-life balance,<br>Time management,<br>Preparation<br>Prioritization.
	Research knowledge and intellectual abilities.	Subject knowledge<br>Theoretical knowledge on research methods<br>Practical application on research methods<br>Information seeking<br>Information literacy and management<br>Academic reading<br>Critical thinking<br>problems solving.
	Research governance and management.	Research management<br>Multimedia management<br>Reference management<br>Financial management<br>Seeking funding

	Engagement, influence, and impact	Seeking a scholarship.<br>Publication,<br>Presentations at conferences<br>Communication<br>Collaboration<br>Team working<br>People management<br>Supervision<br>Teaching
Research completion levels		Generating ideas<br>Background work<br>Preparing, organizing, and collecting data<br>Analyzing data<br>Writing creating, revisiting primary<br>Research output<br>Defending research project or thesis
